# Fluorescence Transduction of Liquid Crystal Ordering Transitions for Biosensing

**DOI:** 10.1021/jacs.5c16679

**Published:** 2026-01-12

**Authors:** Mauricio Vera-Arévalo, Alberto Concellón

**Affiliations:** Instituto de Nanociencia y Materiales de Aragón (INMA), Departamento de Química Orgánica, 16765 CSIC-Universidad de Zaragoza, 50009 Zaragoza, Spain

## Abstract

Liquid crystal (LC) ordering transitions are exquisitely sensitive to molecular interactions at aqueous interfaces and have long served as the basis for optical biosensors. However, the readout of these transitions has almost exclusively relied on polarized-light optical microscopy, which limits quantification and hinders practical deployment. Here, we report a fluorescence-based transduction scheme that converts LC ordering transitions to quantitative optical outputs. Our strategy employs amphiphilic block copolymers bearing aggregation-induced emission (AIE) motifs that undergo dynamic covalent conjugation with IgG antibodies through reversible imine chemistry. In complex LC emulsions, polymer surfactants localize differently depending on droplet LC configuration: accumulation at monopolar defects concentrates AIE units to generate a bright ON state, whereas redistribution along the LC/water interface in the radial configuration suppresses emission to yield an OFF state. Recognition of *Salmonella enterica* serovar Typhimurium—one of the most prevalent foodborne pathogens—reversibly perturbs this equilibrium, producing rapid (∼1 h) ON/OFF fluorescence responses with detection limits down to 10^2^ cells/mL. Incorporation of a ratiometric reference dye further enhances robustness against experimental variability. This work establishes the fluorescence transduction of LC ordering transitions as a generalizable and portable sensing paradigm, bridging soft matter design with real-world diagnostics.

## Graphical abstract

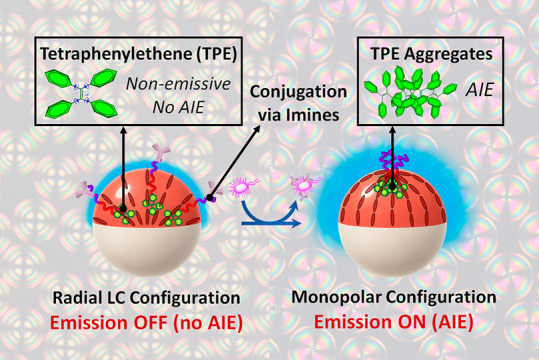

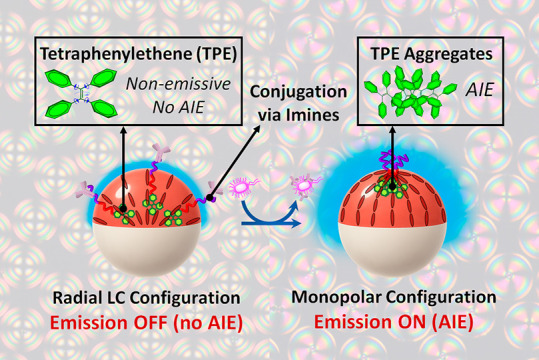

## Introduction

Liquid crystals (LCs) are dynamic soft matter systems that combine long-range orientational order with fluidity.^1^ Their intrinsic anisotropy and responsiveness to external cues have enabled applications ranging from displays to optics, soft robotics, and biosensing.^2–4^ Of particular relevance for sensing, the orientational order of LCs is extremely sensitive to molecular-level interactions at interfaces.^5–8^ Even subtle perturbations, such as the adsorption of biomolecules, can trigger ordering transitions that are readily transduced into mesoscale changes in the LC director field. This amplification mechanism effectively converts interfacial recognition events into macroscopic optical responses.^9,10^

Confinement of LCs into micrometer-sized droplets further enhances this sensitivity.^11,12^ In emulsified geometries, the large interfacial area, the necessity of surfactants, and the emergence of topological defects endow LC droplets with a rich repertoire of configurations that are acutely responsive to interfacial perturbations. Indeed, LC emulsions have been reported to undergo ordering transitions in response to a broad spectrum of analytes, including lipids, proteins, and pathogens.^13–22^ Despite this rich phenomenology, the readout of LC-based biosensors has almost exclusively relied on polarized-light optical microscopy. While this approach has been invaluable for fundamental studies, it hinders quantification, requires specialized equipment, and limits translation into practical platforms. Alternative transduction methods, such as flow cytometry of LC droplets or machine learning applied to scattering patterns, or polarizer-free dye-doped LC sensors^23–25^ have recently emerged, yet a generalizable and portable optical output remains an unmet challenge.

Among LC emulsions, dynamically reconfigurable complex emulsions composed of LCs and immiscible fluorocarbon oils expand the sensing landscape by introducing compartmentalization and topological defects.^26^ These droplets can adopt Janus or core–shell morphologies and undergo reversible transitions in both droplet geometry and internal LC ordering in response to interfacial cues.^27–29^ Such features enable the selective localization of surfactants and recognition elements within nanoscale defect cores, thereby amplifying molecular binding events into mesoscale ordering transitions. Despite these opportunities, their potential in biosensing remains underexplored, and optical readouts have continued to rely on polarized microscopy rather than portable or quantitative methods.

Herein, we introduce a fluorescence-based sensing paradigm that transduces LC ordering transitions into quantitative optical outputs (Figure 1). Our approach exploits amphiphilic block copolymers bearing aggregation-induced emission (AIE) motifs that undergo dynamic covalent conjugation with IgG antibodies through reversible imine formation. Antibody-functionalized Janus droplets adopt a radial configuration in which the polymers are distributed along the LC/water interface, suppressing the AIE and yielding an OFF state. Recognition of *Salmonella enterica* serovar Typhimurium—one of the most prevalent foodborne pathogens—competitively removes IgG from the interface, triggering a transition to a monopolar configuration in which AIE units concentrate at the defect core, thereby producing a bright ON state that directly reports on the LC ordering transition. Beyond *Salmonella*, the modularity of this approach makes it broadly adaptable to other pathogens or biomolecular targets simply by varying the recognition element. This mechanism circumvents the need for polarized-light optical microscopy, provides a quantitative fluorescence readout that can be stabilized through ratiometric referencing, and achieves detection limits down to 10^2^ cells/mL. The simplicity and adaptability of this strategy position complex LC emulsions as practical and versatile biosensors for real-world pathogen detection.

**Figure 1:**
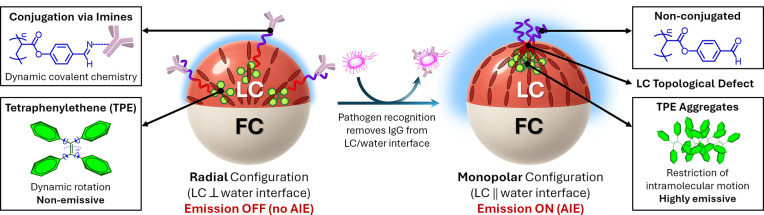
Fluorescence transduction of LC ordering transitions from dynamically reconfigurable complex emulsions, where one compartment is an LC (red bars indicate the LC director field configuration) and the other is a fluorocarbon oil (FC). In the radial configuration, antibody-conjugated polymers distribute uniformly at the LC/water interface, suppressing AIE and producing an emission OFF state. Upon pathogen recognition, IgG is competitively removed from the interface, inducing a monopolar configuration in which polymer surfactants concentrate at the topological defect, promoting TPE aggregation and yielding a bright emission ON state.

## Results and Discussion

Side-chain liquid-crystalline polymer surfactants have long been used to trigger ordering transitions in response to interfacial events, as structural rearrangements at the LC/water boundary propagate through the mesophase and alter its orientational order.^30–37^ Building on this concept, we designed amphiphilic block copolymers consisting of an LC-compatible hydrophobic block and a hydrophilic block bearing aldehyde groups for dynamic covalent conjugation^38^ with recognition elements such as IgG antibodies (Figure 2a). To transduce ordering transitions into a fluorescence output, we incorporated tetraphenylethylene (TPE) motifs into the hydrophobic block. Owing to their aggregation-induced emission (AIE) behavior,^39^ TPE units are nonemissive when dispersed but become strongly fluorescent upon self-assembly, where intramolecular rotations are restricted.

**Figure 2:**
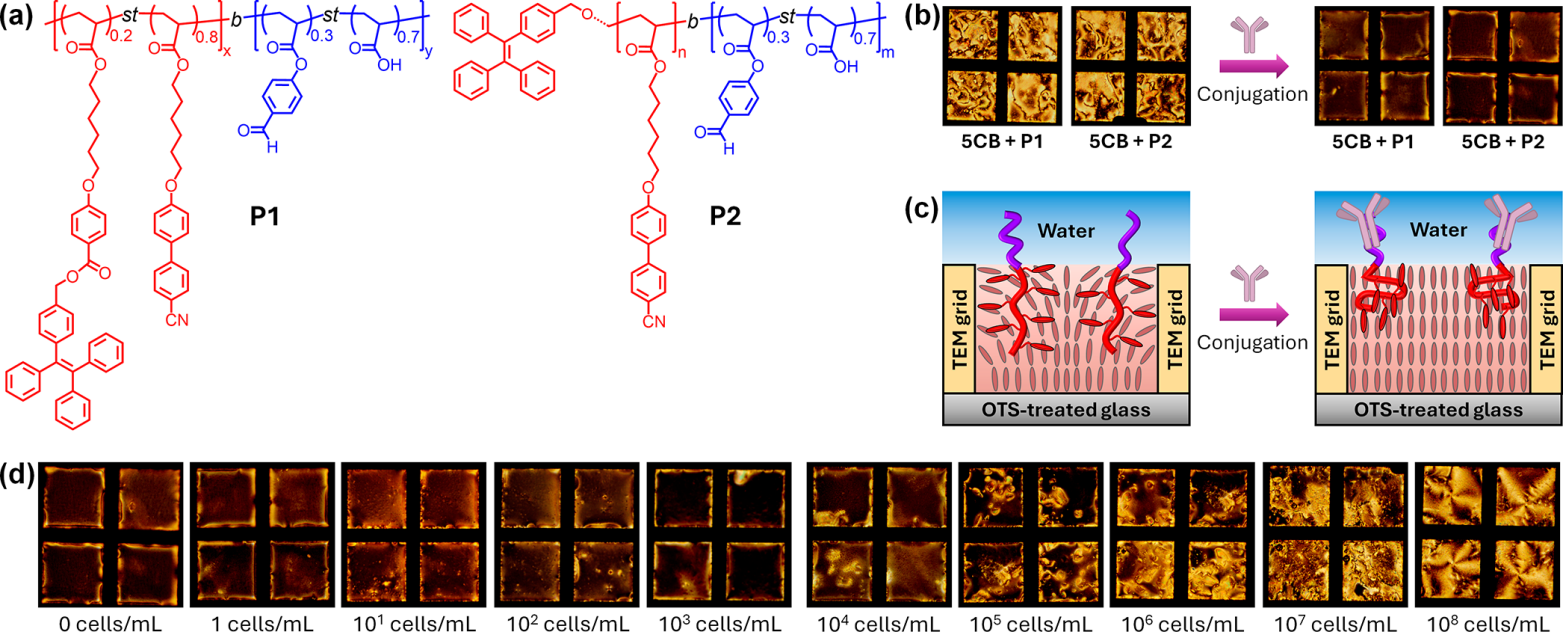
(a) Chemical structures of amphiphilic block copolymers **P1** and **P2** bearing aldehyde groups for dynamic covalent conjugation and TPE motifs for AIE. (b) POM images of **5CB** films containing **P1** or **P2** (5 mg/mL) before and after conjugation with anti-*Salmonella typhimurium* IgG antibodies (35 μg/mL), showing the transition from birefringent to uniformly dark textures consistent with homeotropic anchoring. (c) Schematic illustration and corresponding POM images of **5CB** films confined in TEM grids supported on an OTS-treated glass and immersed in a HEPES buffer solution (10 mM), highlighting the conformational changes of polymer surfactants upon antibody conjugation that promote homeotropic alignment. (d) POM images of IgG-decorated **5CB** films after incubation with increasing concentrations of HKST (1–10^8^ cells/mL), showing the progressive recovery of Schlieren textures due to competitive extraction of IgG from the LC interface.

To assess the influence of polymer architecture, we synthesized two amphiphilic copolymers, **P1** and **P2** (Figure 2a). Both polymers share comparable molecular weights (*M*_n_ ≈ 20 kDa), hydrophilic content (∼20 wt %), and LC block chemistry but differ in the positioning of the TPE motifs. In **P1**, TPE and cyanobiphenyl acrylate units were statistically copolymerized at a 20:80 wt % ratio, a design that minimizes self-AIE while maintaining solubility in the nematic LC. In **P2**, by contrast, a single TPE motif was introduced as a terminal group. The selected molecular weights and composition ratios were guided by prior studies on polymeric amphiphiles at LC interfaces, ensuring both interfacial activity and compatibility with the nematic host.^27,29^ To evaluate their effect on nematic ordering, we prepared thin films of **5CB** doped with **P1** and **P2** in TEM grids supported on an OTS-treated glass to enforce homeotropic anchoring at the solid surface. Pure **5CB** films, when immersed in HEPES aqueous buffer (10 mM, pH 7.4), displayed Schlieren textures under crossed polarizers, indicative of planar anchoring at the aqueous interface (Figure 2b). At polymer concentrations below 5 mg/mL, the Schlieren textures persisted, whereas at higher loadings (≥10 mg/mL) signs of immiscibility became evident, with polymer macrophase separation within **5CB**. The solubility thresholds were comparable for **P1** and **P2**, consistent with their similar compositions and amphiphilic balance. These miscibility limits defined the upper concentration range employed in subsequent experiments.

We next evaluated whether bioconjugation of the hydrophilic aldehyde block affected LC anchoring. Films of **5CB** containing **P1** or **P2** (5 mg/mL) were incubated with anti-*Salmonella typhimurium* IgG antibodies for 1 h, which produced a uniform dark appearance under POM (Figure 2b), consistent with complete homeotropic anchoring. This transition is attributed to conformational changes of the polymer chains upon antibody binding.^32–34^ In the nonconjugated state, the compact hydrophilic block covers only a limited interfacial area, allowing the LC block to penetrate into the nematic matrix and preserve planar anchoring (Figure 2c). Upon conjugation, the expanded coil of the antibody-functionalized block spreads across the interface, forcing the LC block into an oblate configuration with mesogenic side groups oriented perpendicular to the surface, thereby promoting homeotropic alignment (Figure 2c). To further substantiate this interpretation, fluorescence emission of the LC films containing **P1** and **P2** was recorded before and after antibody conjugation (Figure S1). For **P1**, containing pendant TPE moieties statistically distributed along the LC block, the emission intensity increased upon bioconjugation, consistent with enhanced restriction of the side-chain TPE units as the polymer adopts a more compact, oblate conformation at the water interface. In contrast, **P2**, bearing a terminal TPE unit, exhibited a decrease in the fluorescence after conjugation. This opposite trend likely arises because the conformational rigidification that accompanies antibody binding limits the ability of terminal TPE groups from neighboring polymers to interact, thereby reducing interchain aggregation that is accessible in the more flexible, nonconjugated state. These results confirm that antibody conjugation induces conformational reorganization of the polymer surfactants at the LC/water interface, consistent with the anchoring transition observed under POM.

We then probed the response of IgG-decorated LC interfaces to *Salmonella enterica* serovar Typhimurium. LC films were incubated with increasing concentrations of heat-killed *Salmonella* (HKST, 1–10^8^ cells/mL) and equilibrated for 1 h (Figure 2d). At concentrations below 10^3^ cells/mL, the films retained a dark optical appearance consistent with homeotropic anchoring. At 10^4^–10^5^ cells/mL, coexisting bright and dark domains emerged, whereas ≥10^6^ cells/mL induced complete Schlieren textures. These transitions are consistent with antibody-mediated recognition of HKST, followed by the competitive extraction of IgG from the LC interface. As a result, the films revert to their preconjugation state, restoring planar anchoring and birefringence. From these results, the detection limit for HKST in thin films was estimated at 10^4^–10^5^ cells/mL. Control experiments supported the specificity of this response. **5CB** films functionalized with a nonspecific antibody (anti-mouse IgG produced in goat) displayed no detectable changes in optical appearance upon exposure to HKST (Figure S2), confirming that the transition is driven by selective antigen recognition rather than nonspecific adsorption.

We next used **P1** and **P2** to functionalize dynamically reconfigurable complex emulsions composed of immiscible nematic LCs and fluorocarbon oils (FC) in order to evaluate their interfacial assembly. Such emulsions exhibit enhanced sensitivity due to their large surface area and the presence of topological defects. Among Janus morphologies, two representative internal configurations are the radial and the monopolar states (Figure 1). The monopolar configuration features a single point defect at the LC compartment’s pole with a typical size of ∼10 nm in thermotropic nematics, known to selectively concentrate amphiphiles and particles.^14,40–44^ In this framework, **P1** and **P2** are expected to localize differently depending on the LC configuration: in the monopolar state, they accumulate at the defect, producing AIE enhancement through a local concentration of TPE units, whereas in the radial state, they distribute uniformly along the LC/water interface, yielding minimal AIE.

Complex LC droplets functionalized with **P1** and **P2** were prepared by an evaporation-induced phase separation (Figure 3a). Briefly, emulsification of a 1:1:3 volume ratio of 5CB/HFE7200/DCM into a 0.01 wt % aqueous solution of poly­(vinyl alcohol) (PVA) yielded droplets that, after DCM evaporation, formed Janus emulsions with monopolar internal LC configurations. In their native state, the droplets gravity-align with the denser FC compartment at the bottom, enabling top-view optical measurements. Fluorescence spectra were collected by using a fiber-optic spectrophotometer coupled to a 325 nm LED positioned above a monolayer of droplets (Figure 3b). The initial spectra showed an emission maximum at ∼410 nm, characteristic of aggregated TPE units concentrated at the monopolar defect. Upon bioconjugation with IgG antibodies via reversible imine chemistry, the emission intensity decreased markedly, consistent with a monopolar-to-radial transition that redistributes polymer chains along the LC/water interface and suppresses AIE (Figure 3c).

**Figure 3:**
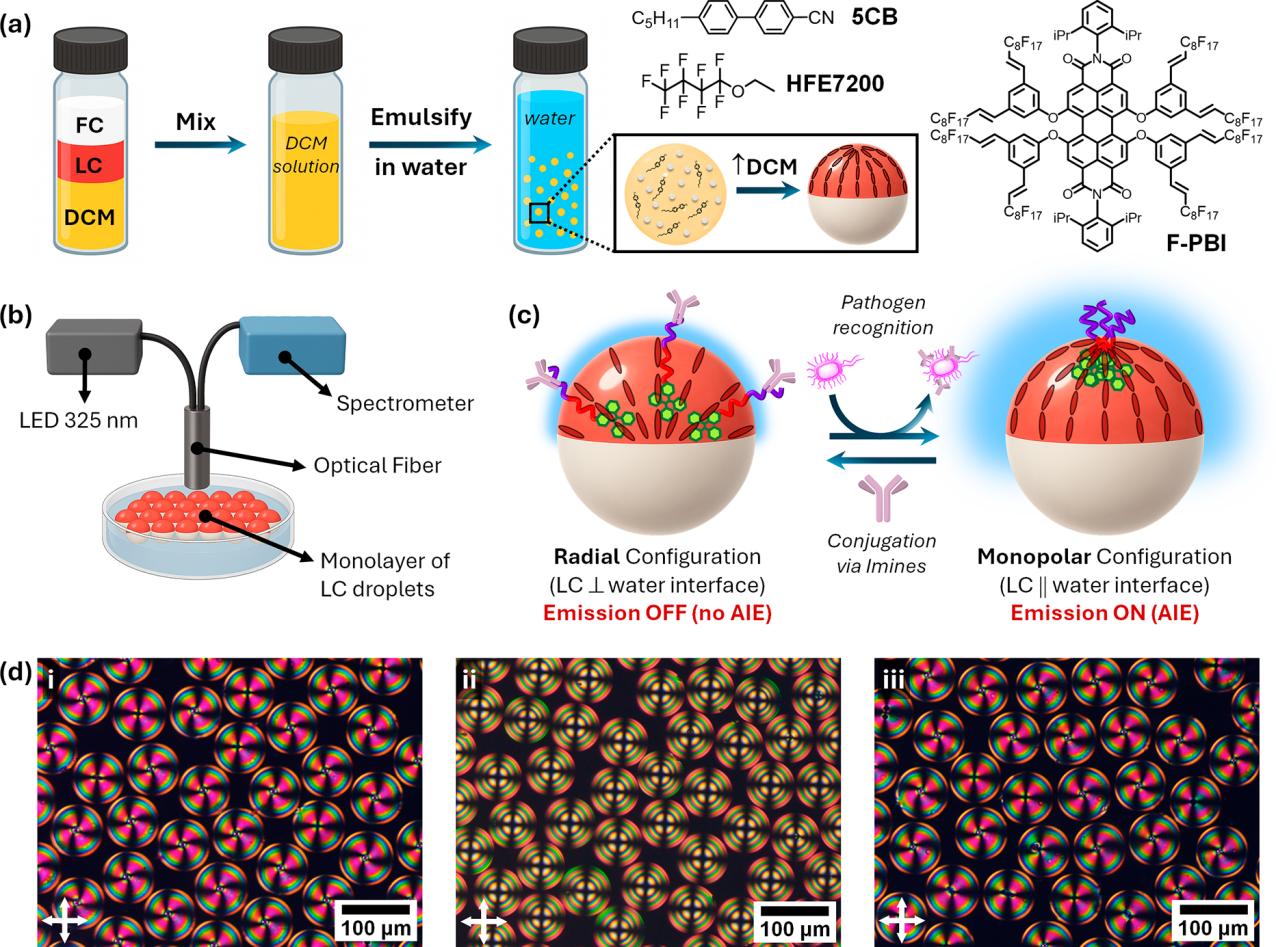
(a) Preparation of complex LC droplets by emulsification of 5CB/HFE7200/DCM in aqueous PVA, followed by DCM evaporation, yielded Janus emulsions with monopolar configurations. Chemical structures of **5CB**, HFE-7200, and the fluorous dye **F-PBI** were used for ratiometric detection. (b) Schematic of the fluorescence measurement setup using a fiber-optic spectrophotometer coupled to a 325 nm LED positioned above a monolayer of droplets. (c) Schematic illustration of the sensing mechanism. (d) Polarized-light optical microscopy images of Janus droplets under crossed polarizers: (i) native state, (ii) after IgG conjugation, and (iii) after incubation for 1 h with *Salmonella enterica* (10^6^ cells/mL).

As in the thin films, the sensing mechanism in emulsions relies on competitive binding and unbinding of IgG antibodies at the LC/water interface (Figure 3c). To evaluate bacterial recognition, IgG-decorated Janus emulsions were incubated with increasing concentrations of HKST (1–10^8^ cells/mL). Emission intensity increased progressively with HKST concentration (Figure 4), reflecting restoration of the monopolar configuration through the competitive removal of IgG from the interface by bacterial binding (Figure 3d). Figure 5 displays the corresponding calibration plot correlating the emission intensity ratio (*I*/*I*_0_) to the concentration of HKST cells. Both **P1**- and **P2**-functionalized emulsions exhibited this behavior but with notable differences in performance. **P2** showed a sharper OFF–ON response: in its antibody-conjugated state, the fluorescence baseline was markedly reduced (though not fully suppressed), yielding a lower background. By contrast, **P1** exhibited partial self-AIE even in the OFF state, leading to a higher fluorescence background. This difference arises from their architectures: in **P1**, TPE units are pendant groups along the LC block, promoting intrachain TPE–TPE interactions and self-aggregation, whereas in **P2** a single terminal TPE motif remains largely isolated until interfacial aggregation occurs. As a result, **P1** shows fluorescence leakage in the OFF state, which reduces the relative signal change upon recognition. Consequently, the detection limit was 10^2^–10^3^ cells/mL for **P2**—substantially lower than that in thin films—whereas **P1** yielded 10^3^–10^4^ cells/mL.

**Figure 4:**
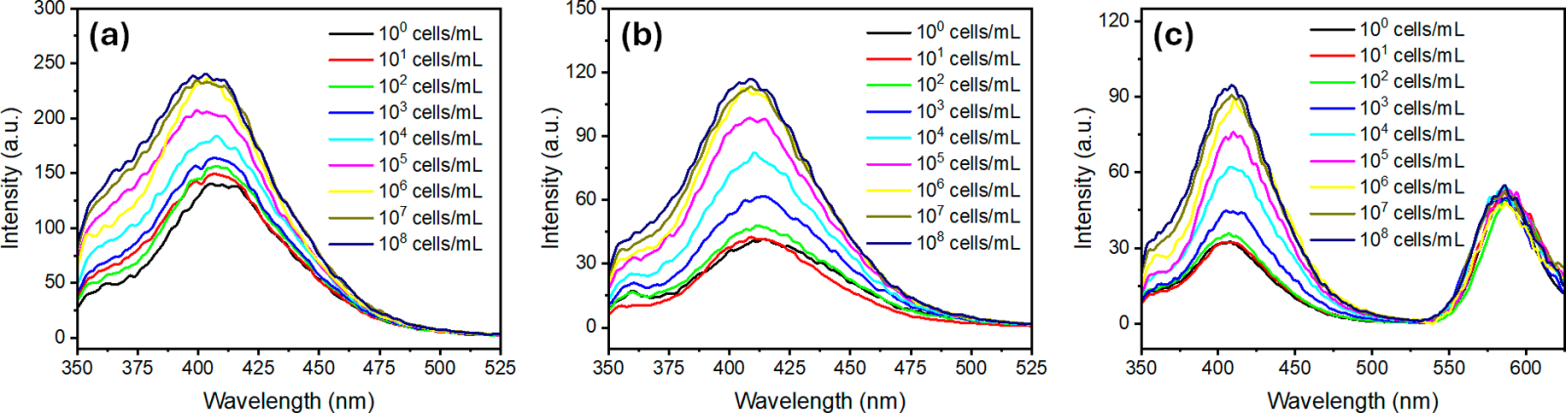
Fluorescence emission spectra (λ_exc_ = 325 nm) of complex LC emulsions upon exposure to increasing concentrations of *Salmonella enterica* (HKST, 1–10^8^ cells/mL): (a) **P1**-functionalized emulsions, (b) **P2**-functionalized emulsions, and (c) **P2**-functionalized emulsions also containing the fluorous reference dye **F-PBI** in the FC compartment for ratiometric detection.

**Figure 5:**
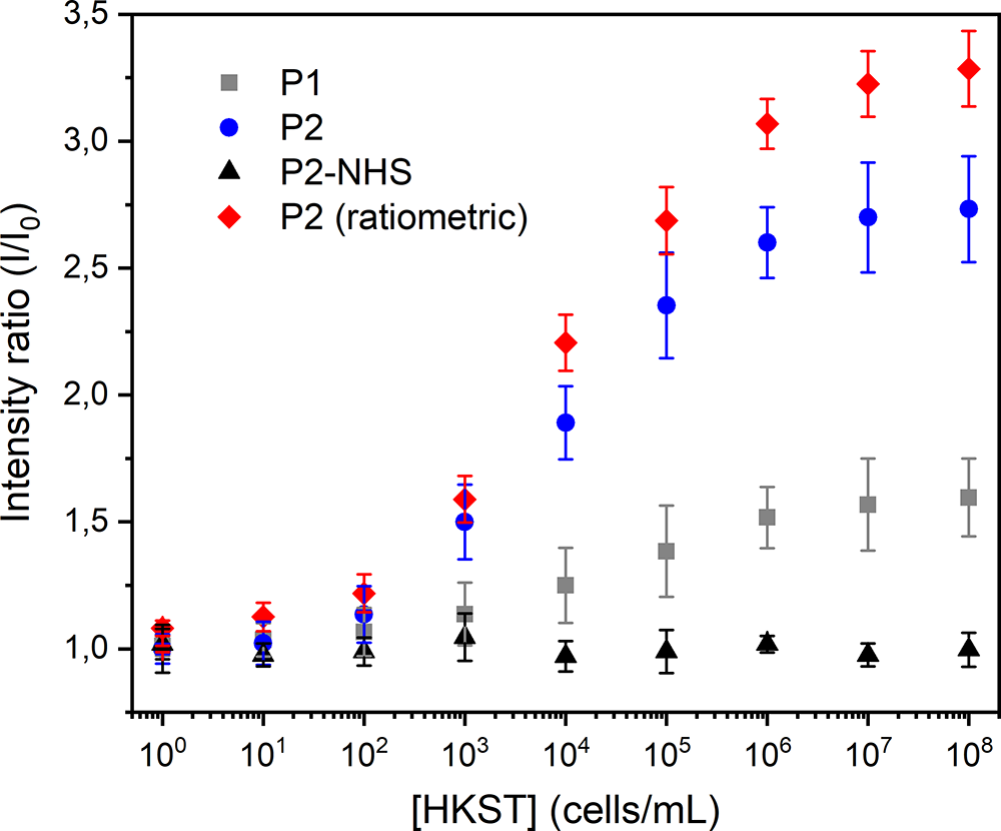
Detection of *Salmonella enterica* using complex LC emulsions. Relative emission intensity (*I*/*I*_0_) as a function of the bacterial concentration. Data are shown as mean ± standard deviation (*N* ≥ 5). Ratiometric emission intensities (*I*_LC_/*I*_FC_) were obtained by dividing the fluorescence of **P2** in the LC phase (λ = 415 nm) by that of the fluorous reference dye **F-PBI** in the FC phase (λ = 580 nm).

Control experiments using an analogous NHS-functionalized **P2** polymer (**P2-NHS**) that forms irreversible amide linkages with IgG confirmed the importance of reversible antibody immobilization. In NHS-based emulsions, no change in emission intensity was observed upon exposure to HKST, consistent with the absence of antibody extraction from the interface (Figure 5). These results highlight that dynamic interfacial chemistry is essential for pathogen-induced optical readouts. In addition, emulsions functionalized with a nonspecific antibody (anti-mouse IgG produced in goat) displayed no measurable change in fluorescence or ratiometric signal upon exposure to HKST (Figure S3), confirming that the ON/OFF response is driven by specific antigen recognition rather than nonspecific adsorption. Moreover, we examined the influence of nontarget proteins by introducing bovine serum albumin (BSA) into the assay medium. At moderate concentrations (≤0.1 mg/mL, ≈1.5 μM), the fluorescence signals of **P2**-based emulsions remained largely unchanged, whereas higher BSA levels led to partial attenuation of the ON response (Figure S3), consistent with competitive interactions of primary amines with the dynamic imine linkages at the LC/water interface. Under these conditions, the antibody is displaced from the interface, rendering the LC emulsions unresponsive to HKST, while the polymer remains conjugated to BSA through dynamic imine bonds that stabilize the initial radial-like configuration and prevent the ordering transition to the monopolar state with defect-localized aggregation of TPEs. Consequently, high concentrations of nontarget proteins can reduce the apparent assay sensitivity; this effect can be mitigated by simple sample dilution or prepurification and, in future designs, by employing less nucleophile-sensitive dynamic bioconjugation linkages.

To improve the reliability of the sensing platform, we implemented a ratiometric scheme by incorporating a second, inert dye into the FC compartment of Janus emulsions.^45,46^ In this configuration, TPE emission from the LC phase (λ ≈ 415 nm) was monitored alongside the reference dye emission from the FC phase (λ ≈ 580 nm) (**F-PBI** in Figure 3a). Calculating the intensity ratio (*I*_LC_/*I*_FC_) normalized variations arising from droplet size distribution, droplet number, excitation power, or optical alignment, thereby enabling reliable measurements even in polydisperse emulsions. As shown in Figure 4, the ratiometric signal paralleled the absolute TPE intensity changes upon antibody binding and subsequent bacterial recognition, but with reduced variability across experiments. Notably, the detection limit of 10^2^–10^3^ cells/mL for **P2** emulsions was maintained, while the assay reproducibility was markedly improved.

These detection limits are competitive with those of state-of-the-art biosensing methods for *Salmonella* detection—including DNA-, enzyme-, and immunoassay-based techniques—that typically require enrichment steps, specialized instrumentation, or long incubation times.^47,48^ In contrast, our fluorescence-based LC emulsion assay provides quantitative results within ∼1 h by using simple optical readouts. The ratiometric design further relaxes the need for monodisperse droplet arrays or microfluidic generation, allowing reliable measurements on randomly deposited emulsions and facilitating a straightforward adaptation to compact or portable fluorimeters. This combination of sensitivity, rapidity, and operational simplicity highlights the versatility and translational potential of fluorescence-transduced LC sensors. Previous LC sensors employing fluorescent dopants have mainly relied on solid film slabs,^6,25^ where dye alignment reports orientation changes of the nematic host, yielding qualitative rather than quantitative optical responses and limited specificity due to nonspecific analyte adsorption at LC interfaces. In contrast, the present system integrates selective bioconjugation with dynamically reconfigurable LC emulsions, enabling specific molecular recognition and reversible ON/OFF fluorescence outputs that quantitatively report analyte-recognition-triggered ordering transitions.

## Conclusions

In conclusion, we have established a fluorescence-based sensing paradigm in which amphiphilic LC polymer surfactants couple interfacial ordering transitions with aggregation-induced emission readouts. By harnessing the confined geometries of Janus LC emulsions, we created ON/OFF fluorescence signals that directly report on LC ordering transitions and enable rapid (∼1 h), sensitive detection of *Salmonella enterica* with limits of detection down to 10^2^ cells/mL with **P2**-based systems. The implementation of a ratiometric readout provides an internal calibration that ensures robustness and strengthens quantitative analysis, relaxing the need for strictly monodisperse emulsions in practical assays. The simplicity and portability of the fluorescence output make this platform competitive with established bacterial detection methods, which typically require lengthy enrichment steps or costly instrumentation. Beyond *Salmonella*, this dynamic emulsion sensor scheme is broadly adaptable to other pathogens or biomolecular targets, positioning complex LC emulsions as practical and versatile biosensing platforms that bridge soft matter design with real-world diagnostics.

## Supplementary Material



## Notes

Materials and characterization techniques, experimental procedures, synthesis and characterization, supplementary figures, NMR spectra (PDF)

## ABBREVIATIONS

5CB: 4-cyano-4′-pentylbiphenyl
AIE: aggregation-induced emission
BSA: bovine serum albumin
DCM: dichloromethane
FC: fluorocarbon oil
HFE-7200: hydrofluoroether 7200 (ethylnonafluorobutyl ether)
HKST: heat-killed *Salmonella enterica* serovar Typhimurium
IgG: immunoglobulin G
LC: liquid crystal
NHS: *N*-hydroxysuccinimide
OTS: octadecyltrichlorosilane
POM: polarized-light optical microscopy
PVA: poly­(vinyl alcohol)
TEM: transmission electron microscopy
TPE: tetraphenylethylene
