# Supplementary material for: Fluorescence Transduction of Liquid Crystal Ordering Transitions for Biosensing
Source: J Am Chem Soc. 2026 Jan 12;148(3):3167–73. doi: 10.1021/jacs.5c16679 (PMC12856913; doi:10.1021/jacs.5c16679)
Supplement: Supplementary file 1 [file ja5c16679_si_001.pdf]

Supporting Information

Fluorescence Transduction of Liquid Crystal Ordering Transitions for Biosensing

*Mauricio Vera-Arévalo, and Alberto Concellón**

Instituto de Nanociencia y Materiales de Aragón (INMA), Departamento de Química Orgánica, CSIC-Universidad de Zaragoza, 50009 Zaragoza, Spain.

** Author for correspondence: aconcellon@unizar.es*

CONTENTS

1. **Materials and Characterization Techniques**

   1.1. Materials

   1.2. Instruments

2. **Experimental Procedures**

   2.1. Preparation of the LC mixtures of 5CB and amphiphilic block copolymers

   2.2. Preparation of LC optical cells for LC/water interface studies

   2.3. General procedure for the preparation of complex LC emulsions

   2.4. Antibody immobilization and *Salmonella* sensing

3. **Synthesis and Characterization**

   3.1. Synthesis of TPE-based Monomer (**1**) and TPE-based CTA (**2**)

   3.2. Synthesis of polymer **P1**

   3.3 Synthesis of polymer **P2** and polymer **P2-NHS**

4. **Supplementary Figures**

5. **NMR spectra**

1. MATERIALS AND CHARACTERIZATION TECHNIQUES

2.1. Materials

1,1,2-Triphenyl-2-[4-(hydroxymethyl)phenyl]ethene,[1] 4-[6-(acryloyloxy)hexyloxy]benzoic acid,[2] 6-(4-cyano-biphenyl-4'-yloxy)hexyl acrylate,[3] and the fluorous perylene bisimide dye (**F-PBI**)[4] were prepared following previously reported procedures. Commercial reagents were used as received without further purification: poly(vinyl alcohol) (PVA, Mw 13000-23000, 87-89% hydrolyzed), tert-butyl acrylate, 4-hydroxybenzaldehyde, 2,2′-azobis(2-methylpropionitrile) (AIBN), 4-cyano-4-((dodecylsulfanylthiocarbonyl)sulfanyl)pentanoic acid, *N,N'*-dicyclohexylcarbodiimide (DCC), anhydrous *N,N*-dimethylformamide (DMF), anti-mouse IgG antibody produced in goat, anti-*Salmonella Typhimurium* antibody, bovine serum albumin (Sigma-Aldrich); 4-cyano-4'-pentylbiphenyl (5CB) (Synthon Chemicals, Germany); 1-(Ethoxy)nonafluorobutane (HFE-7200) (TCI); HEPES buffer (1M) (ThermoFisher); dichloromethane, tetrahydrofuran, methanol, ethyl acetate (Fisher Scientific). Anhydrous dichloromethane and tetrahydrofuran were purchased from Sigma-Aldrich. Deuterated solvents were purchased from Eurisotop. Gold specimen grids (30 μm thickness, 284 × 284 μm hole size, 30 μm bar width) were obtained from Aname.

2.2. Instruments

Fourier-transform infrared (FTIR) spectra were recorded on a Bruker Vertex 70 spectrometer using KBr pellets. Solution-state NMR spectra were acquired on Bruker Avance spectrometers operating at 400 MHz (^1H) and 100 MHz (^{13}C), using standard pulse sequences. Chemical shifts (δ) are reported in ppm relative to tetramethylsilane (TMS), with residual solvent signals used as internal references. Size exclusion chromatography (SEC) was performed on a Waters e2695 Alliance liquid chromatography system equipped with a Waters 2424 evaporative light-scattering detector using two Styragel® columns (HR4 and HR1, Waters). Measurements were carried out in THF at a flow rate of 1 mL/min, using poly(methyl methacrylate) (PMMA) narrow-dispersity standards for calibration. Elemental analysis was performed on a PerkinElmer 2400 microanalyzer. MALDI-TOF mass spectrometry was conducted on a Bruker Autoflex instrument using dithranol as the matrix. Liquid-crystal films and emulsions were examined by polarized-light optical microscopy (POM) on an Olympus BH-2 polarizing microscope equipped with a Linkam THMS600 hot stage. Fluorescence emission spectra were recorded on an Avantes AvaSpec-ULS2048 spectrometer coupled to a fiber-optic reflection probe and a 325 nm LED excitation source from Mightex.

---

[1] *Polym. Chem.* **2014**, *5*, 3758-3762.
[2] *Adv. Funct. Mater.* **2019**, *29*, 1905214
[3] *Macromolecules* **1995**, *28*, 3617-3624.
[4] *Synlett* **2018**, *29*, 2509-2514.

2. EXPERIMENTAL PROCEDURES

2.1. Preparation of the LC mixtures of 5CB and amphiphilic block copolymers

Dichloromethane (DCM) solutions containing 5CB and the desired amount of amphiphilic block copolymer were allowed to slowly evaporate at room temperature. The resulting mixtures were further dried under vacuum.

2.2 Preparation of LC optical cells for LC/water interface studies

A detailed description of the optical cell preparation can be found elsewhere.[5] Briefly, clean glass slides were activated by cold plasma using a PiezoBrush PZ3 device, followed by coating with octadecyltrichlorosilane (OTS) by immersion in a 0.5 mM OTS solution in heptane for 30 min at room temperature, rinsed with dichloromethane, and dried under vacuum. These grids were placed on the OTS-coated glass, and 1 µL of 5CB was deposited onto each grid. Excess 5CB was removed by gently contacting the droplet with a capillary tube, leading to the formation of a stable film of 5CB within the grid. The surface of the 5CB film in contact with the aqueous phase was approximately flat, as verified by the concurrent focusing of the grid and LC film under an optical microscope. The OTS-treated substrates containing 5CB films were immersed into 500 µL of aqueous solution to form stable LC/water interfaces.

2.3. General procedure for the preparation of complex LC emulsions

Complex emulsions were prepared either by bulk emulsification or using a microfluidic device to obtain polydisperse or monodisperse droplets, respectively. Both approaches yielded droplets with well-defined morphology and composition.

In a typical bulk emulsification procedure, 35 µL of the dispersed phase (LC/HFE-7200/DCM, 1:1:3 v/v) was added to 500 µL of a 0.1 wt. % aqueous PVA solution. The mixture was vortexed at 4000 rpm for 10 s, followed by slow evaporation of DCM at room temperature to induce phase separation within the droplets. For homogenization, droplets were heated to the isotropic state and then cooled to room temperature prior to use. This procedure produced polydisperse droplets with diameters ranging from 5 to 200 µm, as observed by optical microscopy.

For microfluidic preparation, emulsions were generated using a Droplet Junction Chip (100 µm, Dolomite Microfluidics) with two Mitos P pressure pumps to independently control the dispersed and continuous phases. The fluids were pressurized with compressed air, providing stable, pulseless flow to the flow-focusing chip (pressures: dispersed phase, 200 mbar; continuous phase, 500 mbar). This method yielded monodisperse droplets with an average diameter of 80±10 µm, as determined by optical microscopy.

[5] *Langmuir* **2002**, *18*, 6101-6109.

2.4. Antibody immobilization and *Salmonella* sensing

For the preparation of aldehyde-functionalized LC emulsions, **P1** or **P2** block copolymer (2 mg/mL) was dissolved in the dispersed phase (LC/HFE-7200/DCM, 1:1:3 v/v). Droplets were fabricated in 0.1 wt% PVA aqueous solution using the emulsification procedures described above. A surfactant solution was prepared by dissolving PVA in HEPES buffer (10 mM, pH 7.5) at a concentration of 0.05 wt%. Anti-*Salmonella* typhimurium IgG antibodies were pre-dispersed (35 µg/mL) in 1 mL of this surfactant solution and subsequently mixed with 40 µL of aldehyde-functionalized droplets. The emulsions were gently agitated (120 rpm) overnight at room temperature. Prior to measurements, the aqueous surfactant solution was exchanged three times to remove unbound IgG.

For sensing experiments with heat-killed Salmonella enterica serovar Typhimurium (HKST), 40 µL of antibody-functionalized monodisperse droplets were incubated with HKST cells at concentrations ranging from 1 to 10^8 cells/mL. Samples were agitated (120 rpm) for 3 h at room temperature. Measurements were performed using a Thermo Fisher Invitrogen Attofluor Cell Chamber. The glass surface of the chamber was pre-wetted with surfactant solution (500 µL) prior to depositing droplets (40 µL) into the center of the chamber. Droplets settled under gravity, spreading into a monolayer. Reflection spectra were recorded with an Avantes AvaSpec-ULS2048 spectrometer coupled to a fiber-optic reflection probe and a 325 nm LED excitation source. The fiber was positioned 5 cm above the droplet monolayer. For each sample, five spectra were acquired at different positions, and the procedure was repeated three times for each HKST concentration.

3. SYNTHESIS AND CHARACTERIZATION

3.1. Synthesis of TPE-based Monomer (1) and TPE-based CTA (2)

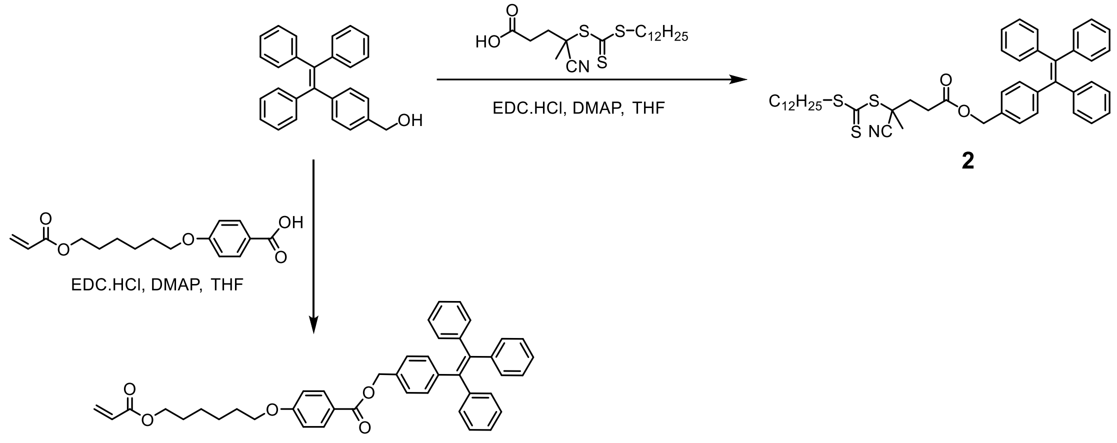

TPE-based monomer (1). 4-[6-(Acryloyloxy)hexyloxy]benzoic acid (1.69 g, 5.52 mmol), 1,1,2-triphenyl-2-[4-(hydroxymethyl)phenyl]ethene (2.00 g, 5.52 mmol), and 4-(dimethylamino)pyridine (0.34 g, 2.77 mmol) were dissolved in anhydrous THF (25 mL). The reaction flask was cooled in an ice bath, flushed with argon, and 1-ethyl-3-(3-dimethylaminopropyl)carbodiimide hydrochloride (1.16 g, 6.07 mmol) was added. The mixture was stirred at room temperature (RT) for 24 h under argon. The resulting white precipitate was filtered off and washed with THF. The solvent was evaporated, and the crude product was purified by flash column chromatography on silica gel (hexane/DCM 1:1 to 1:2). Yield: 62%. IR (ν, cm^{-1}): 3051, 2929, 1725, 1635, 1610, 1567, 1493, 1249, 1197, 1137. ^{1}H NMR ($CDCl_3$, 400 MHz, δ, ppm): 7.98-7.90 (m, 2H), 7.19-6.94 (m, 19H), 6.75-6.68 (m, 2H), 6.44-6.34 (m, 1H), 6.16-6.06 (m, 1H), 6.85-6.78 (m, 1H), 5.27-5.16 (m, 2H), 4.17 (t, *J*= 6.7 Hz, 2H), 3.99 (t, *J*= 6.5 Hz, 2H), 1.86-1.76 (m, 2H), 1.75-1.67 (m, 2H), 1.55-1.39 (m, 2H). ^{13}C NMR ($CDCl_3$, 100 MHz, δ, ppm): 166.96, 166.47, 162.37, 144.11, 144.06, 143.92, 143.52, 141.68, 140.94, 135.17, 133.24, 131.67, 131.57, 131.54, 130.52, 129.05, 128.10, 128.06, 128.02, 127.68, 126.86, 126.83, 126.81, 121.85, 117.73, 111.75, 68.31, 66.14, 64.82, 29.41, 28.94, 26.10, 26.05, 22.46. MS (MALDI$^+$, DIT): m/z calcd. for $C_{44}H_{42}O_5$ $[M+Na]^+$, 673.3; found, 673.2. Anal. calcd. for $C_{44}H_{42}O_5$: C, 81.20%; H, 6.51%. Found: C, 80.96%; H, 6.75%.

TPE-based CTA (2). 4-[6-(Acryloyloxy)hexyloxy]benzoic acid (250 mg, 0.62 mmol), 4-cyano-4-((dodecylsulfanylthiocarbonyl)sulfanyl)pentanoic acid (225 mg, 0.62 mmol), and 4-(dimethylamino)pyridine (38 mg, 0.31 mmol) were dissolved in anhydrous THF (7.5 mL). The solution was cooled in an ice bath, flushed with argon, and 1-ethyl-3-(3-dimethylaminopropyl)carbodiimide hydrochloride (131 mg, 0.68 mmol) was added. The mixture was stirred at RT for 24 h under argon. The resulting precipitate was filtered off, washed with THF, and the solvent was evaporated. The crude product was purified by flash column chromatography on silica gel (hexane/DCM 1:1 to 1:2). Yield: 75%. IR (ν, cm^{-1}): 3044, 2917, 1739, 1597, 1495, 1442, 1179, 1068, 698. ^{1}H NMR (CD_2Cl_2, 400 MHz, δ, ppm): 7.20-6.96 (m, 19H), 5.08-4.98 (m, 2H), 3.40-3.30 (m, 2H), 2.68-2.58 (m, 2H), 2.56-2.45 (m, 1H), 2.43-2.32 (m, 2H), 1.84 (s, 3H),

1.76-1.58 (m, 2H), 1.46-1.17 (m, 18H), 0.88 (t, *J*= 6.9 Hz, 3H). ^{13}C NMR (CD_2Cl_2, 100 MHz, δ, ppm): 218.10, 171.59, 144.26, 144.17, 144.05, 144.01, 141.80, 140.82, 139.40, 134.21, 131.73, 131.66, 131.55, 131.53, 128.13, 128.08, 128.03, 127.93, 126.91, 126.87, 126.85, 126.80, 126.61, 119.41, 66.85, 65.17, 46.86, 37.55, 34.09, 32.32, 30.13, 30.03, 29.95, 29.83, 29.75, 29.47, 29.30, 28.08, 25.03, 23.10. MS (MALDI$^+$, DIT): m/z calcd. for $C_{46}H_{53}NO_2S_3$ [M+Na]$^+$, 770.3; found, 770.5. Anal. calcd. for $C_{46}H_{53}NO_2S_3$: C, 73.85%; H, 7.14%; N, 1.87%, S, 12.86%. Found: C, 73.56%; H, 7.00%; N, 2.07%, S, 13.01%.

3.2. Synthesis of polymer P1

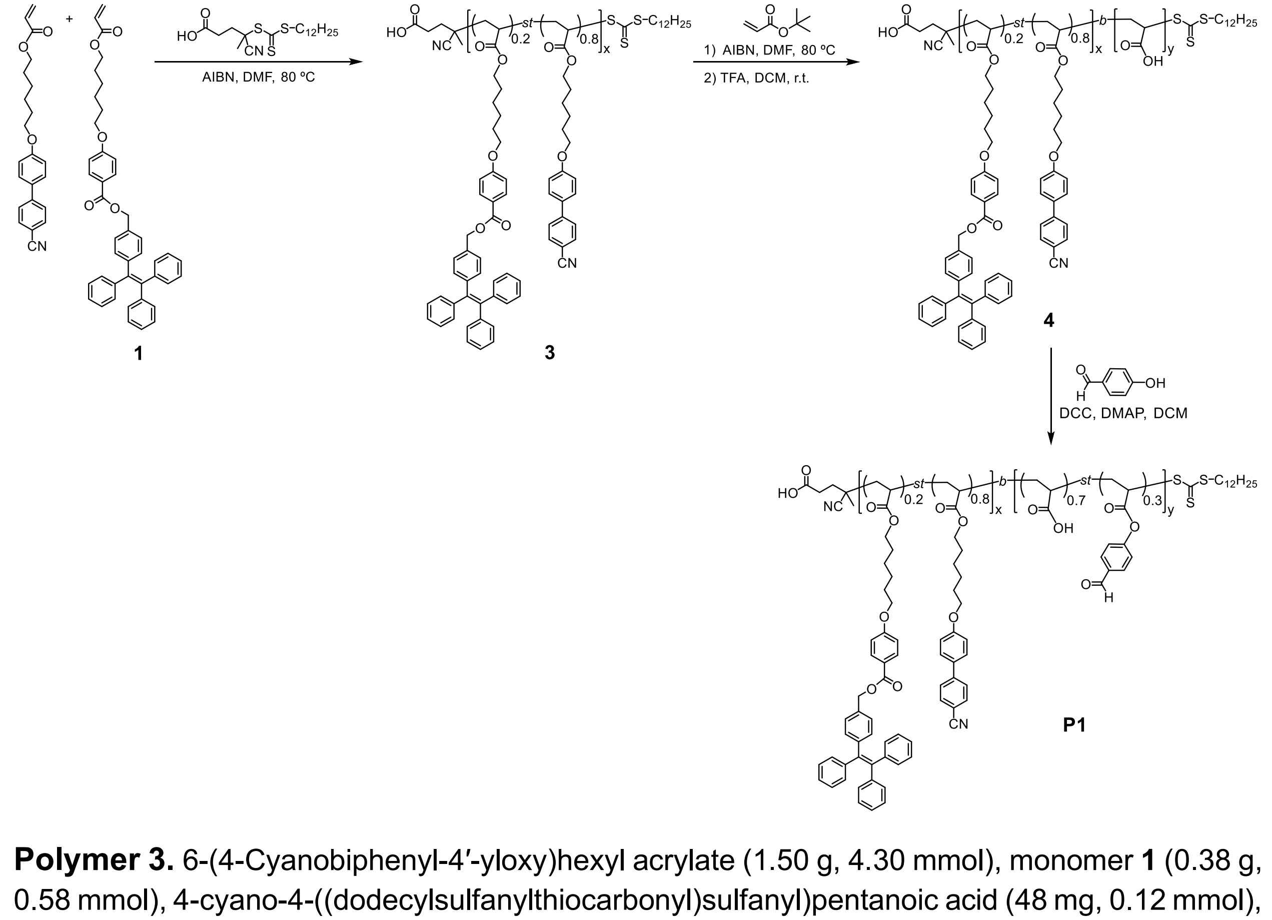

Polymer 3. 6-(4-Cyanobiphenyl-4′-yloxy)hexyl acrylate (1.50 g, 4.30 mmol), monomer **1** (0.38 g, 0.58 mmol), 4-cyano-4-((dodecylsulfanylthiocarbonyl)sulfanyl)pentanoic acid (48 mg, 0.12 mmol), AIBN (3.0 mg, 0.02 mmol), and DMF (5 mL) were added to a Schlenk flask sealed with a rubber septum. The mixture was deoxygenated by three freeze–pump–thaw cycles, flushed with argon, and stirred at 80 °C for 24 h. The reaction was quenched with liquid nitrogen, diluted with THF, and precipitated into cold methanol. The polymer was dried under vacuum at 40 °C for 48 h to give a yellow waxy solid. Yield: 92%. IR (KBr) ν (cm^{-1}): 3049, 2922, 2224, 1738, 1598, 1490, 1442, 1182, 1068, 700. ^{1}H NMR (CD_2Cl_2, 400 MHz, δ, ppm): 7.93-7.79 (m, 7H), 7.76-7.35 (m, 181H), 7.22-6.80 (m, 138H), 6.75-6.56 (m, 10H), 5.25-5.06 (m, 9H), 4.24-3.77 (m, 140H), 3.39-3.24 (m, 2H), 2.62-1.10 (m, 465H). SEC (PS standards): M_n= 13.2 kDa, *Đ*= 1.28.

Polymer 4. Tert-butyl acrylate (0.25 g, 1.95 mmol), polymer 3 (0.50 g), AIBN (1.64 mg, 0.01 mmol), and DMF (2.5 mL) were added to a Schlenk flask. The solution was deoxygenated (three freeze–pump–thaw cycles), flushed with argon, and stirred at 80 °C for 48 h. The mixture was quenched with liquid nitrogen and precipitated into cold methanol. The polymer was dried under vacuum at 40 °C for 48 h to give a yellowish waxy solid. Yield: 69%. IR (KBr) ν (cm^{-1}): 3044, 2933, 2225, 1728, 1604, 1491, 1250, 1157, 821. ^{1}H NMR (CD_2Cl_2, 400 MHz, δ, ppm): 7.92-7.78 (m, 0.25H), 7.74-7.34 (m, 6H), 7.20-6.78 (m, 4.62H), 6.73-6.57 (m, 0.38H), 5.21-5.06 (m, 0.35H), 4.18-3.75 (m, 5H), 2.52-1.07 (m, 25H). SEC (PS standards): M_n= 15.3 kDa, $Đ$= 1.27.

Then, the resulting polymer (0.3 g) was dissolved in anhydrous DCM (15 mL) with trifluoroacetic acid (3 mL and stirred at RT for 24 h. The solution was concentrated under reduced pressure and precipitated into cold methanol. The polymer was dried under vacuum at 40 °C for 48 h to give a white solid. Yield: 83%. IR (KBr) ν (cm^{-1}): 3429, 3049, 2927, 2225, 1730, 1602, 1492, 1491, 1247, 1169, 821. ^{1}H NMR (CD_2Cl_2, 400 MHz, δ, ppm): 8.00-7.77 (m, 0.5H), 7.75-7.32 (m, 6H), 7.17-6.78 (m, 4.22H), 6.74-6.57 (m, 0.55H), 5.21-5.08 (m, 0.35H), 4.22-3.74 (m, 5H), 2.61-1.05 (m, 20H). SEC (PS standards): M_n= 15.8 kDa, $Đ$= 1.29

Polymer P1. Polymer **4** (0.25 g), 4-hydroxybenzaldehyde (0.10 g, 0.82 mmol), and 4-(dimethylamino)pyridine (25 mg, 0.20 mmol) were dissolved in anhydrous THF (10 mL). The solution was cooled in an ice bath, flushed with argon, and N,N′-dicyclohexylcarbodiimide (0.19 g, 1.0 mmol) was added. The mixture was stirred at RT for 48 h under argon. The precipitate was filtered off and washed with THF. The solvent was evaporated, and the crude product was precipitated twice into cold methanol. The polymer was dried under vacuum at 40 °C for 48 h, affording **P1** as a white powder. Yield: 43 IR (KBr) ν (cm^{-1}): 3045, 2929, 2224, 1729, 1605, 1493, 1249, 1159, 821. ^{1}H NMR (CD_2Cl_2, 400 MHz, δ, ppm): 10.11-10.05 (m, 0.2H), 8.00-7.77 (m, 0.38H), 7.75-7.32 (m, 6H), 7.17-6.78 (m, 4.62H), 6.74-6.57 (m, 0.55H), 5.21-5.08 (m, 0.35H), 4.22-3.74 (m, 5H), 2.61-1.05 (m, 20H). SEC (PS standards): M_n= 15.5 kDa, $Đ$= 1.31.

3.3. Synthesis of polymer P2 and polymer P2-NHS

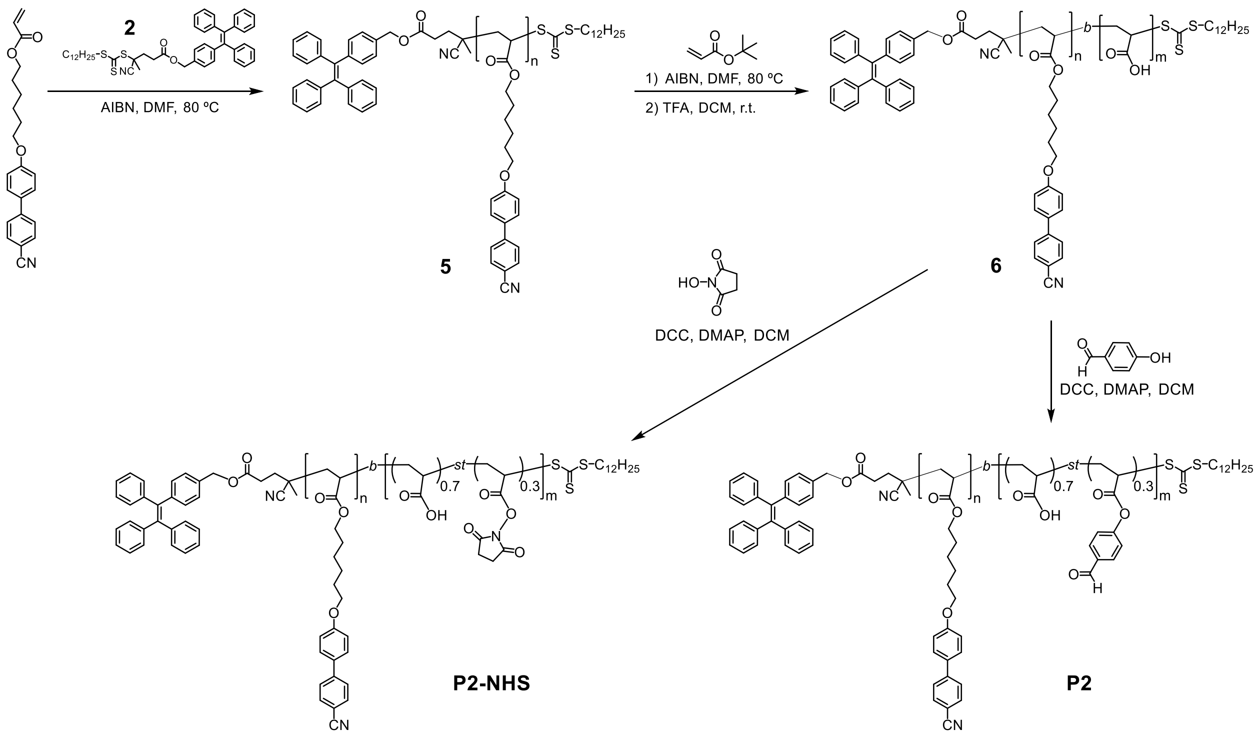

Polymer 5. 6-(4-Cyanobiphenyl-4′-yloxy)hexyl acrylate (1.50 g, 4.30 mmol), 4-cyano-4-((dodecylsulfanylthiocarbonyl)sulfanyl)pentanoic acid (64.2 mg, 0.09 mmol), AIBN (2.8 mg, 0.017 mmol), and DMF (5 mL) were added to a Schlenk flask sealed with a rubber septum. The mixture was deoxygenated by three freeze–pump–thaw cycles, flushed with argon, and stirred at 80 °C for 24 h. The reaction was quenched with liquid nitrogen, diluted with THF, and precipitated into cold methanol. The polymer was dried under vacuum at 40 °C for 48 h to give a yellow waxy solid. Yield: 65%. IR (KBr) ν (cm^{-1}): 3054, 2938, 2225, 1732, 1672, 1602, 1491, 1248, 1171, 821. ^{1}H NMR (CD_2Cl_2, 400 MHz, δ, ppm): 7.73-7.34 (m, 319H), 7.14-6.76 (m, 124H), 5.00-4.90 (m, 2H), 4.15-3.75 (m, 214H), 3.36-3.21 (m, 2H), 2.51-1.13 (m, 640H). SEC (PS standards): M_n= 10.8 kDa, $Đ$= 1.33.

Polymer 6. Tert-butyl acrylate (0.25 g, 1.95 mmol), polymer **5** (0.50 g), AIBN (1.64 mg, 0.01 mmol), and DMF (2.5 mL) were added to a Schlenk flask. The solution was deoxygenated (three freeze–pump–thaw cycles), flushed with argon, and stirred at 80 °C for 48 h. The mixture was quenched with liquid nitrogen and precipitated into cold methanol. The polymer was dried under vacuum at 40 °C for 48 h to give a yellowish waxy solid. Yield: 56%. IR (KBr) ν (cm^{-1}): 3060, 2938, 2225, 1728, 1601, 1493, 1253, 1159, 822. ^{1}H NMR (CD_2Cl_2, 400 MHz, δ, ppm): 7.75-7.35 (m, 6H), 7.13-6.76 (m, 2.4H), 4.19-3.77 (m, 4H), 2.52-1.12 (m, 21H). SEC (PS standards): M_n= 12.3 kDa, $Đ$= 1.35.

Then, the resulting polymer (0.3 g) was dissolved in anhydrous DCM (15 mL) with trifluoroacetic acid (3 mL and stirred at RT for 24 h. The solution was concentrated under reduced pressure and precipitated into cold methanol. The polymer was dried under vacuum at 40 °C for 48 h to give a white solid. Yield: 83%. IR (KBr) ν (cm^{-1}): 3419, 3046, 2933, 2220, 1732, 1601, 1495, 1247, 1170,

819. ^{1}H NMR (CD_2Cl_2, 400 MHz, δ, ppm): 7.77-7.32 (m, 6H), 7.13-6.77 (m, 2.4H), 4.19-3.77 (m, 4H), 2.57-1.08 (m, 15H). SEC (PS standards): M_n= 13.0 kDa, *Đ*= 1.32

Polymer P2. Polymer **6** (0.25 g), 4-hydroxybenzaldehyde (0.10 g, 0.82 mmol), and 4-(dimethylamino)pyridine (25 mg, 0.20 mmol) were dissolved in anhydrous THF (10 mL). The solution was cooled in an ice bath, flushed with argon, and N,N′-dicyclohexylcarbodiimide (0.19 g, 1.0 mmol) was added. The mixture was stirred at RT for 48 h under argon. The precipitate was filtered off and washed with THF. The solvent was evaporated, and the crude product was precipitated twice into cold methanol. The polymer was dried under vacuum at 40 °C for 48 h, affording **P2** as a white powder. Yield: 62%. IR (KBr) ν (cm^{-1}): 3422, 3058, 2935, 2225, 1729, 1604, 1498, 1251, 1161, 821. ^{1}H NMR (CD_2Cl_2, 400 MHz, δ, ppm): 10.13-10.06 (m, 0.3H), 7.77-7.32 (m, 6H), 7.13-6.77 (m, 2.3H), 4.19-3.77 (m, 4H), 2.57-1.08 (m, 15H). SEC (PS standards): M_n= 13.4 kDa, *Đ*= 1.33.

Polymer P2-NHS. Polymer **6** (0.10 g) and *N*-hydroxysuccinimide (0.05 g, 0.43 mmol) were dissolved in anhydrous THF (5 mL). The reaction flask was cooled in an ice bath and flushed with argon, then *N,N′*-dicyclohexylcarbodiimide (0.11 g, 0.52 mmol) was added. The mixture was stirred at RT for 48 h under argon atmosphere. The white precipitate was filtered off and washed with THF. The solvent was evaporated and the crude product was carefully precipitated twice using cold methanol. The polymer was dried in a vacuum oven at 40 °C for 48 h, obtaining **P2-NHS** as white powder. Yield: 53%. IR (KBr) ν (cm^{-1}): 3426, 3054, 2935, 2224, 1726, 1603, 1496, 1250, 1166, 821. ^{1}H NMR (CD_2Cl_2, 400 MHz, δ, ppm): 7.75-7.30 (m, 6H), 7.12-6.77 (m, 2H), 4.20-3.77 (m, 4H), 2.59-1.10 (m, 15H). SEC (PS standards): M_n= 13.3 kDa, *Đ*= 1.31.

4. SUPPLEMENTARY FIGURES

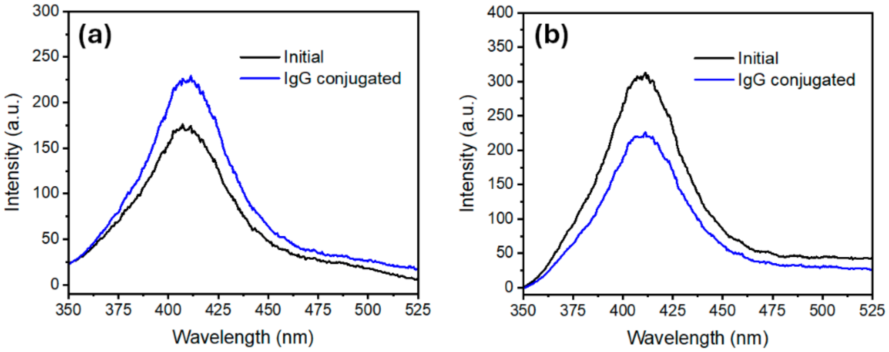

Figure S1. Fluorescence emission spectra of LC films containing **(a) P1** and **(b) P2** (5 mg/mL in 5CB) before (black) and after (blue) conjugation with anti-*Salmonella Typhimurium* IgG antibodies (35 µg/mL).

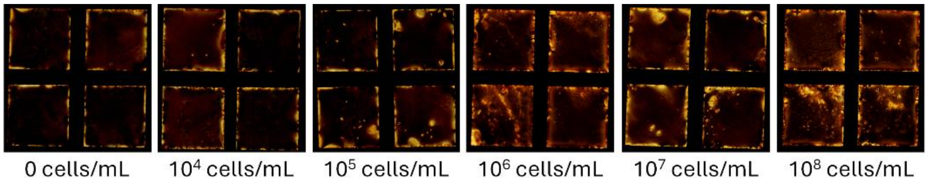

Figure S2. POM images of 5CB films functionalized with a non-specific IgG antibody (anti-mouse IgG produced in goat) after incubation with increasing concentrations of HKST (0–10^8 cells/mL). No change in optical texture is observed upon exposure to HKST, confirming the absence of nonspecific antigen-induced anchoring transitions.

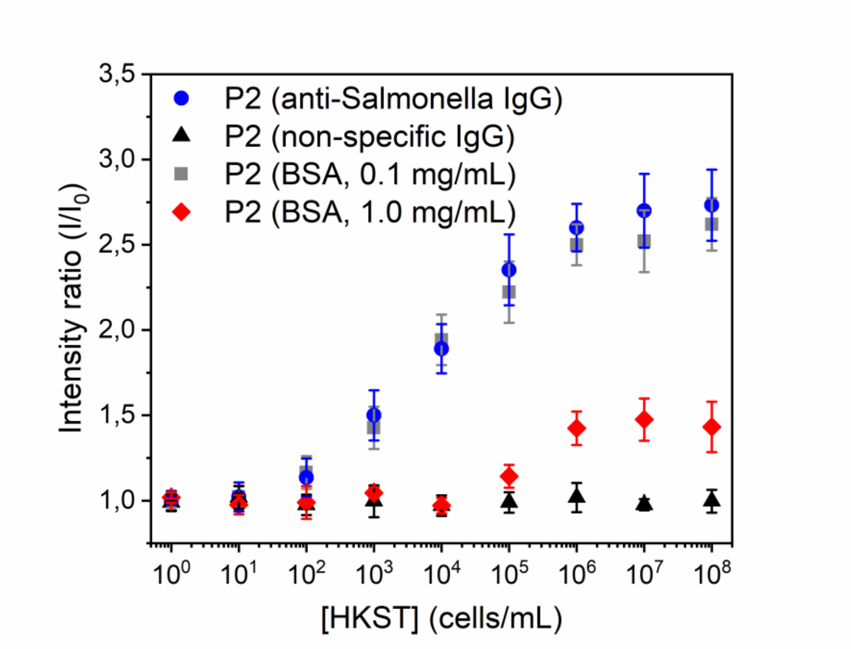

Figure S3. Relative emission intensity (I/I_0) as a function of bacterial concentration for **P2**-based LC emulsions functionalized with anti-*Salmonella Typhimurium* IgG, a non-specific IgG antibody (anti-mouse IgG produced in goat), and in the presence of bovine serum albumin (BSA). Data are shown as mean ± standard deviation (N ≥ 5).

5. NMR SPECTRA

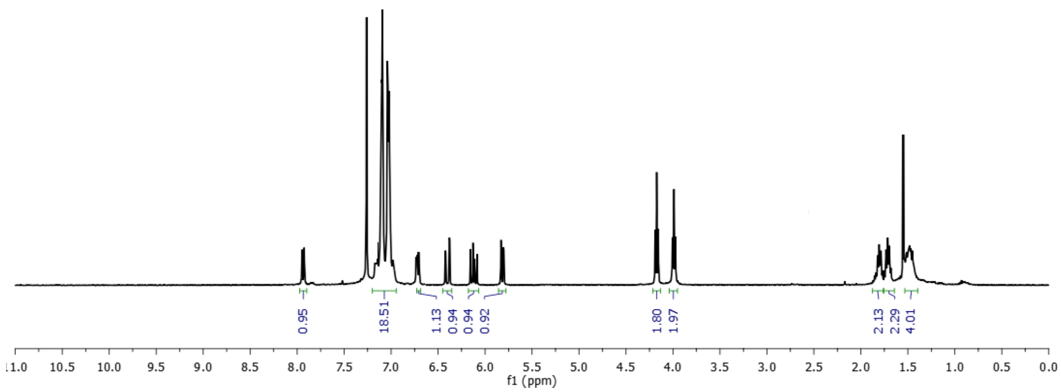

Figure S4. ^{1}H NMR spectrum (400 MHz, $CDCl_3$) of TPE-based monomer (**1**).

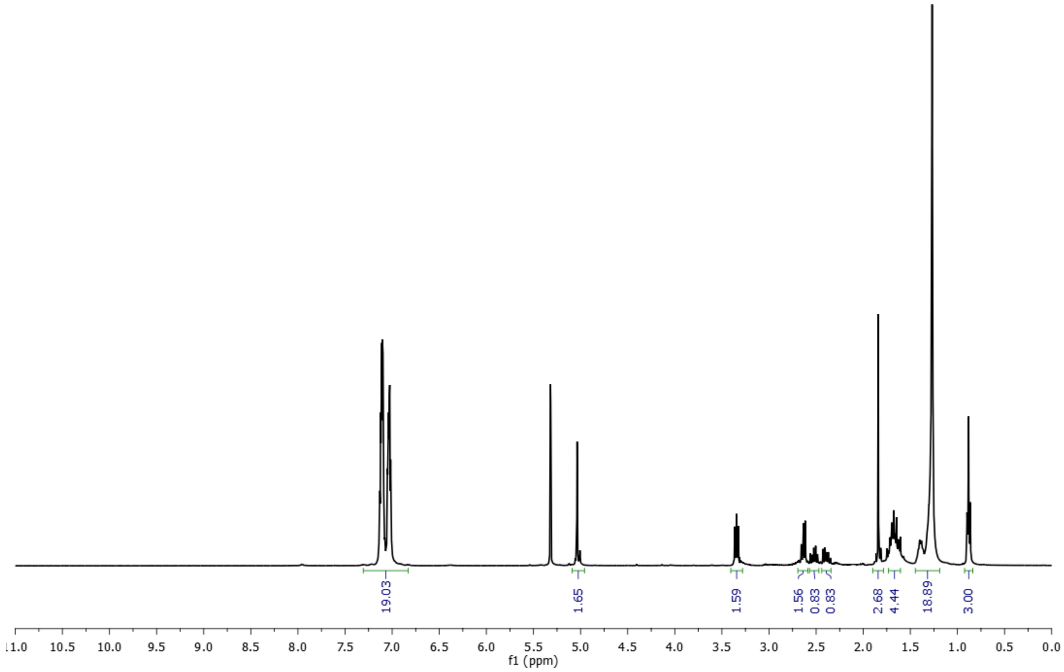

Figure S5. ^{1}H NMR spectrum (400 MHz, CD_2Cl_2) of TPE-based CTA (**2**).

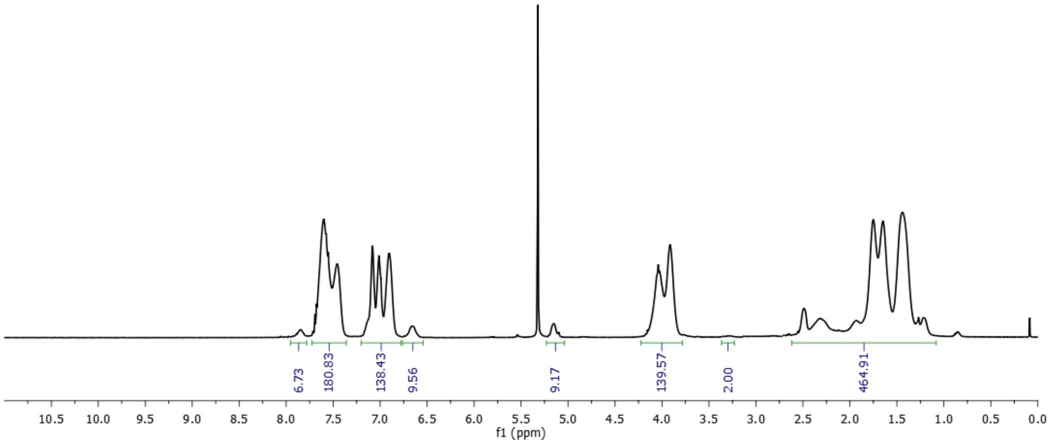

Figure S6. ^{1}H NMR spectrum (400 MHz, CD_2Cl_2) of polymer **3**.

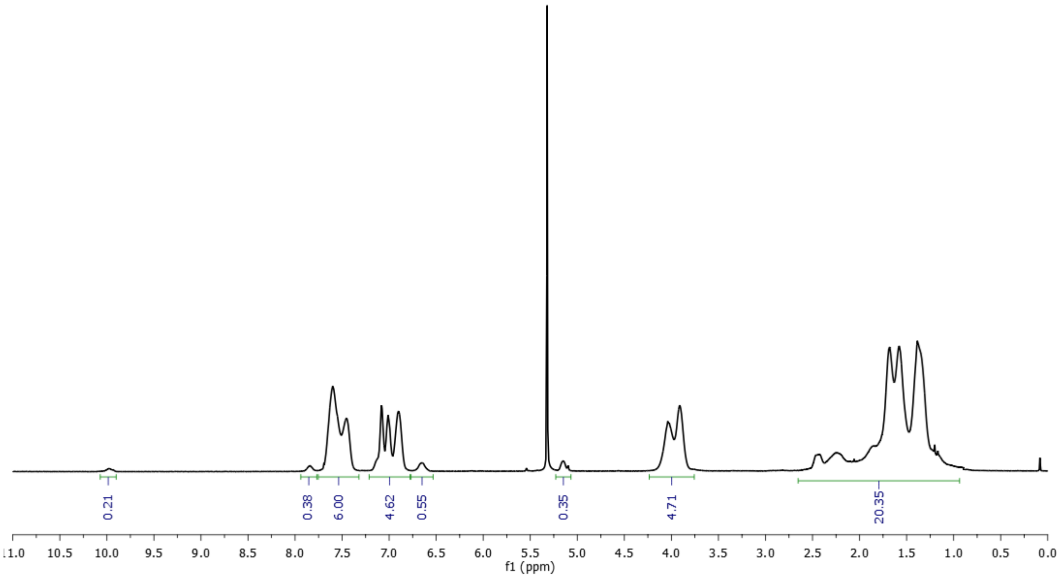

Figure S7. ^{1}H NMR spectrum (400 MHz, CD_2Cl_2) of polymer **P1**.

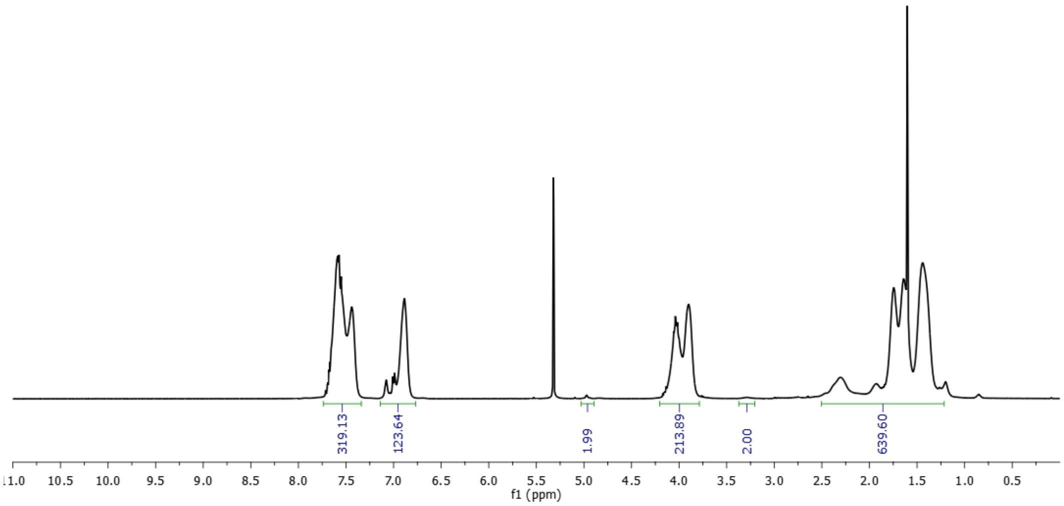

Figure S8. ^{1}H NMR spectrum (400 MHz, CD_2Cl_2) of polymer **5**.

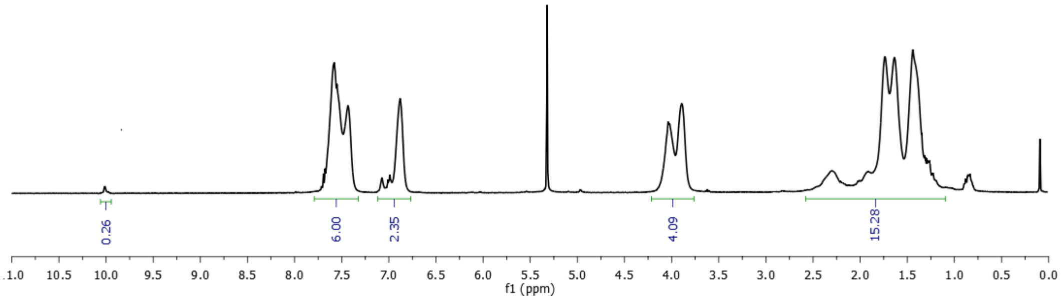

Figure S9. ^{1}H NMR spectrum (400 MHz, CD_2Cl_2) of polymer **P2**.

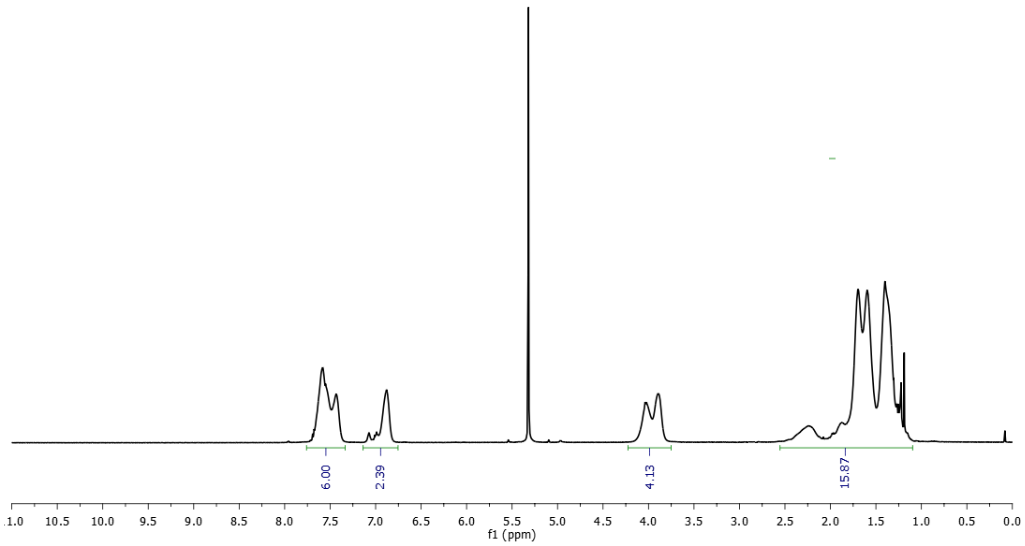

Figure S10. ^1H NMR spectrum (400 MHz, CD_2Cl_2) of polymer **P2-NHS**.
